# Comparison of inflammatory cells, C-reactive protein, and lipid profile in atherosclerotic cardiovascular disease patients and healthy controls in Northwest Ethiopia

**DOI:** 10.1038/s41598-025-21319-5

**Published:** 2025-10-27

**Authors:** Minyahl Kassye Mengstu, Getu Girmay, Kassaye Demeke Altaye, Wossen Habtu, Yoseph Tolcha, Emiyamrew Getnet, Mulualem Lemma, Wubet Birhan

**Affiliations:** 1 Department of Medical Laboratory Sciences, College of Medicine and Health Sciences, Arba Minch University, Arba Minch, Ethiopia; 2 Department of Immunology and Molecular Biology, School of Biomedical and Laboratory Science, College of Medicine and Health Sciences, University of Gondar, Gondar, Ethiopia; 3 Department of Emergency and Critical Care Medicine, College of Medicine and Health Sciences, University of Gondar, Gondar, Ethiopia; 4 Department of Clinical Chemistry, Ethiopian Public Health Institute, Addis Ababa, Ethiopia; 5 Department of Medical Laboratory Sciences, College of Medicine and Health Sciences, Wollo University, Dessie, Ethiopia

**Keywords:** Atherosclerotic cardiovascular disease, Inflammatory cells, C-reactive protein, Lipid profile, Inflammatory cells ratio, Systemic inflammation, Northwest ethiopia, Biomarkers, Cardiology, Diseases, Medical research

## Abstract

Atherosclerotic cardiovascular disease (ASCVD) remains a leading cause of global mortality and disability. Inflammation is increasingly recognized as a key driver in the pathogenesis of ASCVD. However, data on the specific distribution of inflammatory cells, high-sensitivity C-reactive protein (hsCRP), and lipid profiles among ASCVD patients in this region remain limited. Therefore, we aimed to assess the distribution of inflammatory cells, hsCRP, and lipid profile among ASCVD patients and healthy controls. A comparative cross-sectional study was conducted from June to November 2024 at the University of Gondar Comprehensive Specialized Hospital, recruiting 101 ASCVD patients and 101 healthy controls using simple random sampling. Sociodemographic, behavioral, and clinical data were collected using a structured questionnaire, measurement, and chart review. Six milliliters of fasting venous blood were drawn for complete blood count (CBC), hsCRP, and lipid profile analyses. Data were analyzed using STATA version 14. Means were compared using independent t-tests and ANOVA at a significance level of *p* ≤ 0.05 with a 95% confidence interval. Of the 202 study participants, 101 were ASCVD patients and 101 were healthy controls. The mean ages were 57.42 ± 12.88 years for ASCVD patients and 56.30 ± 10.93 years for healthy controls. ASCVD patients had higher mean WBC and neutrophil counts, and higher median monocytes, NLR, MLR, hsCRP, TC, and LDL (*p* ≤ 0.05) than controls, while median eosinophil levels were significantly lower (*p* ≤ 0.05). Our study showed that ASCVD patients had higher inflammatory cell counts, ratios (NLR, MLR), hsCRP, total cholesterol, and LDL than controls, highlighting the potential roles of inflammation and dyslipidemia in ASCVD development.

**Supplementary Information:**

The online version contains supplementary material available at 10.1038/s41598-025-21319-5.

## Introduction

Cardiovascular diseases (CVD), defined as disorders of the heart and blood vessels account for a significant proportion of non-communicable disease (NCD) mortality, with at least 19 million deaths recorded in 2021^1^. In Africa, CVD contributes 38.3% to the continent’s overall NCD mortality^2^, with prevalence in Eastern Sub-Saharan Africa ranging from 19.5% to 42.9%^3^. In Ethiopia, CVDs have become a major public health concern^4^ due to the growing burden of risk factors such as diabetes mellitus, hypertension, dyslipidemia, chronic inflammatory diseases, smoking, family history, and other social behaviors^5^.

Atherosclerotic cardiovascular disease (ASCVD) is a chronic artery disease caused by the buildup of lipoproteins and cholesterol in the arterial wall^6^. The pathogenesis of ASCVD is a complex interaction of lipid metabolism and inflammation^7–9^ in which, at the early stage, atherosclerosis involves endothelial injury, causing expression of adhesion molecules and decreased endothelial progenitor cells on the endothelial wall that causes the infiltration of Low density lipoprotein (LDL) and immune cells to endothelium^10^.

Many studies^11–16^ showed that LDL causes the development of ASCVD as oxidative stress causes LDL to be oxidized into oxidized-LDL (ox-LDL), which is recognized as a damage-associated molecular pattern and engulfed by macrophages to form foam cells, which play a crucial role in forming atherosclerotic plaques^17^. The plaque becomes covered by a fibrous cap and calcified to be stable^18^. Over time, the plaque becomes unstable, rupture/erode, initiating coagulation processes that lead to acute coronary syndrome (ACS) and stroke^19^.

The common immune cells involved in ASCVD are neutrophils, monocytes, macrophages, and platelets^20^. Neutrophils within the endothelial intima release granule proteins that promote monocyte recruitment, inflammation, foam cell formation, and atherosclerosis progression^21^. The recruited monocytes differentiate into macrophages or dendritic cells that phagocytose ox-LDL, forming foam cells^22^. Macrophages also release interferon-gamma (IFN-γ) and matrix metalloproteinase-9 (MMP-9), which cause apoptosis of vascular smooth muscle cells, leading to plaque erosion or rupture^23^. Lymphocytes promote the development of ASCVD through the production of atherogenic cytokines^24^. Platelets also play a crucial role in ASCVD by interacting with ox-LDL^25^, and their coagulation cascade contributes to atherothrombotic complications^26^.

C-reactive protein (CRP), an acute-phase inflammatory protein, can be used as a biomarker to predict the development of ASCVD^27^ and plays a direct role in all stages of the atherosclerotic process^28^. Elevated CRP levels disrupt endothelial function, cause vascular cell activation, induce expression of endothelial receptors for ox-LDL, lipid accumulation, and thrombosis^29^.

Several studies^30,31^ showed the role of inflammation in the development of ASCVD. However, to the best of our knowledge, there is limited understanding about the distribution of inflammatory cells, highly sensitive C-reactive protein (hsCRP), and lipid profile in ASCVD patients in the study area with the rising burden of ASCVD in Ethiopia. Assessing the distribution of inflammatory cells, hsCRP, and lipid profile is essential for early diagnosis, risk assessment, and potential therapeutic interventions. Thus, this study aimed to evaluate the distribution of inflammatory cells, hsCRP, and lipid profile among ASCVD patients and healthy controls at the University of Gondar Comprehensive Specialized Hospital (UoG-CSH).

## Methods

### Study design, period, and setting

An institution-based comparative cross-sectional study was conducted from 20 June to 15 November 2024 at the UoG-CSH. The UoG-CSH is one of the largest teaching hospitals in Ethiopia, with more than 450 beds for inpatients. The hospital provides different inpatient and outpatient health services for more than 7.5 million people of the Central Gondar Zone and neighboring regions. The UoG-CSH provides different health services for the community, such as diagnosis, treatment, management, and follow-up of non-communicable diseases like CVD, as well as communicable diseases^32^.

### Source and study populations

All individuals visiting the UoG-CSH and diagnosed with ASCVD served as the source population for the case group, while individuals who visited the Central Gondar Blood Bank for blood donation constituted the source population for the control group. The study population for the case group included all newly diagnosed ASCVD patients, confirmed by CT scan, MRI, echocardiography (ECHO), or electrocardiography (ECG), who were above 18 years of age, admitted to UoG-CSH during the study period, and who voluntarily provided informed consent. In addition, sex and age-matched healthy controls and who voluntary gave informed consent were considered as a comparator groups in the current study.

**Fig. 1:**
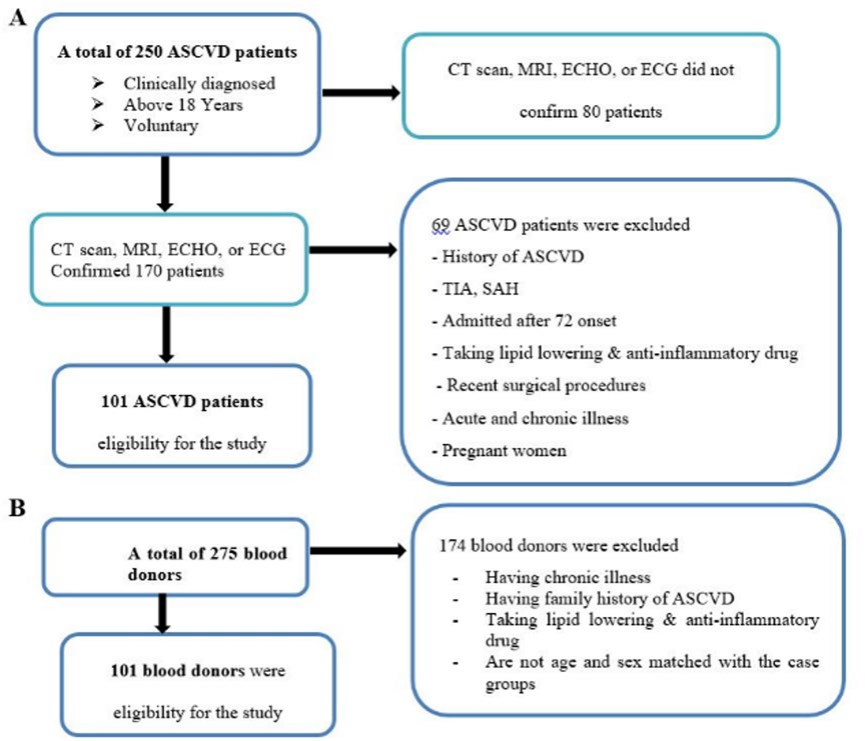
Flow chart of the recruitment and enrollment process of study participants. Newly diagnosed ASCVD patients were recruited from the emergency ward, stroke unit, and inpatient unit of UoG-CSH Gondar, Ethiopia (**A**). Healthy control blood donors were recruited from the central Gondar blood bank, Gondar, Ethiopia (**B**).

To avoid the effects of prior medications, ASCVD patients with a history of the disease were excluded. Additionally, individuals diagnosed with transient ischemic attack (TIA), hemorrhagic stroke, or subarachnoid hemorrhage (SAH) were not included, as these conditions are not always caused by atherosclerosis. Also, individuals who were admitted more than 72 h after the onset of ASCVD, those taking lipid-lowering medications, steroidal or non-steroidal anti-inflammatory drugs, those with a recent history of surgical procedures, known acute or chronic illnesses (except hypertension and diabetes mellitus), or who were pregnant were excluded from this study. Similarly, healthy controls who had a history of any known chronic diseases, had a family member living with or died of ASCVD, those taking lipid-lowering medications, steroidal or non-steroidal anti-inflammatory drugs, as well as any form of sign or symptom of disease during the data collection period were not included in this study (Fig. 1).

### Sample size and sampling technique

The sample size was calculated by using G-power software version 3.1.9.7 (University of Düsseldorf, Germany) with t-test family, for differences between two independent means, and a priori: compute required sample size given α, power, and effect size. A two-tailed and calculated effect size, along with a power of 80%, an α-error probability of 0.05, and an equal allocation ratio (N2/N1 = 1). A 40% effect size was estimated based on the mean and standard deviation of LDL levels reported in a study conducted at Dessie Comprehensive Specialized Hospital^16^. In that study, the mean LDL level for ASCVD cases was 114.07 mg/dL (± 35.82), while for controls it was 103.14 mg/dL (± 15.65). Finally, the total sample size was 202 (101 participants in each group). The study participants were recruited using a simple random sampling technique using the lottery method.

### Data collection

All relevant socio-demographic, clinical, and behavioral data were collected using a pre-tested structured questionnaire and chart review. A well-trained clinical nurse professional working at the UoG-CSH emergency ward, stroke unit, and inpatient unit collected data of ASCVD groups, while a well-trained nurse professional working at the Central Gondar Blood Bank service collected data of healthy controls.

### Operational definition

#### ASCVD status

A condition that occurs when an individual has a diagnosis of atherosclerotic cardiovascular disease (ACS or ischemic stroke) or not.

#### Inflammatory cells

Various immune cells involved in the inflammatory process, including neutrophil, lymphocyte, monocyte, eosinophil, and basophil.

#### Lipid profile

Lipid panel test that measures the level of total cholesterol, triglyceride, low-density lipoprotein, and high-density lipoprotein.

### Blood sample collection and processing

Six milliliters (6 mL) of fasting venous blood were collected from each study participant by laboratory technologists after the site was cleaned with 70% alcohol. Of this, 2 mL were transferred into an EDTA tube and sent to the UoG-CSH hematology laboratory for complete blood count (CBC) analysis. The remaining 4 mL were placed into a serum separator tube (SST), allowed to clot at room temperature for 30 min, and then centrifuged at 3,500 revolutions per minute (RPM) for 5 min to separate the serum. The separated serum was analyzed for lipid profile at the UoG-CSH clinical chemistry laboratory and for high-sensitivity C-reactive protein (hsCRP) at the Ethiopian Public Health Institute (EPHI).

### Differential white blood cell and platelet count

Complete blood count was performed by Mindray BC 5150 (Mindray Bio-Medical Electronics Co., Ltd., Shenzhen, China) using a combination of flow cytometry (FCM), tri-angle laser scatter, and chemical dye methods for WBC quantification and differential analysis, while the platelet count was done through the impedance method^33^. The inflammatory cells were obtained from the CBC result. In addition, the NLR, MLR, and PLR were calculated by dividing the absolute counts of neutrophils, monocytes, and platelets, respectively, by the absolute lymphocyte count obtained from the CBC. The analyzer is factory-calibrated by the manufacturer, with recalibration required only if background or control results fall outside the normal range. When necessary, calibration can be performed using standard, blood samples, and manual calibration mode.

### Determination of TC, TG, LDL, and HDL levels

The quantification of lipid profiles (TC, TG, LDL, and HDL) was performed using a Beckman Coulter DxC 700 AU (Beckman Coulter, Brea, CA, USA) analyzer through the principle of an enzymatic reaction. Calibration of the analyzer was carried out according to the manufacturer’s instructions: for TC and TG, calibration was performed every 30 days, while for LDL and HDL, calibration was performed every 7 days. Recalibration has been conducted whenever there was a change in reagent lot number, a shift in control values was observed, major preventive maintenance was performed on the analyzer, or a critical part was replaced.

The level of serum TC was determined following the incubation of the sample with cholesterol esterase to form cholesterol. Then, cholesterol oxidase was added to the reaction mixture to convert cholesterol to cholest-4-en-3-one and hydrogen peroxide (H_2_O_2_). Then, 4-aminoantipyrine, phenol, and peroxidase were added to the reaction mixture to convert H_2_O_2_ into a chromophore (colored complex). The quantification of TG was performed through the incubation of the sample with lipase to produce glycerol. Then, glycerol kinase and magnesium acetate were added to convert glycerol into glycerol-3-phosphate. The glycerol-3-phosphate was converted to H_2_O_2,_ and the addition of 4-aminoantipyrine (4-AAP), phenol, and peroxidase converts H_2_O_2_ into a colored complex. Finally, the colored complex formed from TC and TG was measured spectrophotometrically at 540/600 and 660/800nm wavelengths, respectively. The increase in absorbance was proportional to the level of TC and TG in the sample.

The levels of serum LDL and HDL were determined after treating the serum samples with detergents that solubilize non-LDL and non-HDL cholesterol, respectively, resulting in a colorless product. Specific detergents were added to release cholesterol from LDL and HDL. Subsequently, cholesterol esterase and cholesterol oxidase were used to produce H_2_O_2_ from the released cholesterol. Finally, the conversion of H_2_O_2_ to colored complexes was mediated by adding DSBmT, 4-AAP, and peroxidase. The colored complex obtained from LDL and HDL was measured at 540/660 and 600/700 nm wavelengths, respectively. The increased absorbance was proportional to the concentration of LDL and HDL in the serum^34^.

### Quantification of highly sensitive C-reactive protein

The level of serum C-reactive protein (CRP) was determined using the Cobas c501 analyzer (Roche Diagnostics, Indianapolis, IN, USA) based on the principle of a particle-enhanced immunoturbidimetric assay. Two commercially prepared reagents, such as R1 (a TRIS buffer containing bovine serum albumin and immunoglobulins) and R3 (latex particles coated with monoclonal anti-CRP antibodies in glycine buffer), were used to analyze CRP. Firstly, the serum sample was incubated with R1 reagent, and R3 reagent was added to the reaction mixture. Then, the reaction mixture was incubated at 37 °C for 10 min. Following incubation, the level of precipitation produced due to the aggregates of CRP with the latex particles was determined using a turbidity measurement assay with a lower detection limit of 0.30 mg/L at 546 nm wavelength. The degree of turbidity (light scattering) was proportional to the amount of CRP in the sample.

The analyzer was calibrated after each reagent lot change and whenever required according to quality control procedures. Calibration intervals could be extended if acceptable verification of calibration results was achieved by the laboratory.

### Data management and quality control

Data quality was maintained in every step of the data collection, and data was collected by well-trained clinical nurses with the supervision of the principal investigator. A pretested structured questionnaire was used to collect sociodemographic and other relevant data. The blood pressure measurements were taken in duplicate, and the average value was used for the analysis. The pre-analytical, analytical, and post-analytical phases of laboratory procedures were followed during the sample processing. In addition, sample processing and analysis were performed as per the standard operating procedures (SOP) and strictly following the manufacturer’s instructions.

### Data processing and analysis

Data was checked for its completeness, coded, and entered into Epi Info 7.2.5.0 (Centres for Disease Control, Georgia, USA). Then, the data was exported to STATA version 14.0 (StataCorp LLC, Texas, USA) for analysis. The normality of the data was assessed using a histogram and the Shapiro-Wilk test. Continuous variables were summarized using means with standard deviations or medians and interquartile ranges, depending on their distribution. Categorical variables were presented using frequencies and percentages. Comparison of inflammatory cells, inflammatory cells ratio, hsCRP, and lipid profile among ASCVD (ASCVD groups) patients and healthy controls was computed using the independent samples t-test and (ANOVA followed by post hoc tests) for normally distributed data. Mann-Whitney U test and Kruskal-Wallis test (followed by Dunn’s test) were utilized for variables that were not normally distributed. A p-value of ≤ 0.05 with a 95% confidence interval was considered statistically significant.

### Ethical statement

The study was conducted after obtaining an ethical approval letter from the Ethical Review Committee of the School of Biomedical and Laboratory Sciences (Reference number: SBMLS/758/2024). A permission letter was obtained from the UoG-CSH medical director. A signed written informed consent was obtained from study participants after the objective of the study was explained to them. In addition, confidentiality of the study participants was maintained by using unique codes for each participant. This study was done as per the Declaration of Helsinki’s ethical guidelines for human studies.

## Results

### Socio-demographic and clinical characteristics of study participants

In this study, 202 study participants with 101 newly diagnosed ASCVD patients and 101 healthy controls (HCs) were included. The majority (52.5%) of the study participants for both ASCVD patients and HCs were males. The mean with SD age of study participants was 57.42 ± 12.88 and 56.30 ± 10.93 years for ASCVD patients and HCs, respectively. Around 60.4% of ASCVD patients and 93.1% of HCs were residing in urban areas (Table 1).

**Table 1:** Sociodemographic and clinical characteristics of study participants from June to November 2024 at UoG-CSH Northwest Ethiopia.

Variable	Total (*N* = 202)	ASCVD (*N* = 101)	HCs (*N* = 101)	*P*-value
Sex^a^				
Female	96 (47.52%)	48 (47.5%)	48 (47.5%)	1.00
Male	106 (52.48%)	53 (52.5%)	53 (52.5%)	
Age (years)^b^	56.87 ± 11.93	57.42 ± 12.88	56.30 ± 10.93	0.51
Residences^a^				
Urban	146 (72.28%)	52 (51.5%)	94 (93.1%)	< 0.001**
Rural	56 (27.72%)	49 (48.5%)	7 (6.9%)	
Marital status^a^				
Single	15 (7.43%)	3 (3.0%)	12 (11.9%)	< 0.001**
Married	140 (69.31%)	63 (62.4%)	77 (76.2%)	
Divorced	11 (5.45%)	5 (5.0%)	6 (6.0%)	
Widowed	36 (17.82%)	30 (29.7%)	6 (6.0%)	
Educational status^a^				
No formal education at all	73 (36.14%)	58 (57.4%)	15 (14.9%)	< 0.001**
Primary	34 (16.83%)	22 (21.8%)	12 (11.9%)	
Secondary	37 (18.32%)	9 (8.9%)	28 (27.7%)	
Collage & above	58 (28.71%)	12 (11.9%)	46 (45.5%)	
Smoking status^a^				
Never	187 (92.57%)	86 (85.2%)	101 (100%)	< 0.001**
Yes	15 (7.43%)	15 (14.9%)	0 (0%)	
Alcohol drinking status^a^				
Never	69 (34.16%)	29 (28.7%)	40 (39.6%)	0.103
Yes	133 (65.84%)	72 (71.3%)	61 (60.4%)	
Physical activity^a^				
Yes	164 (81.19%)	63 (62.3%)	101 (100%)	< 0.001**
Sedentary	38 (18.81%)	38 (37.6%)	0 (0%)	
Family history of ASCVD ^a^				
Yes		24 (24.0%)	NA	
No		76 (76.0%)	NA	
SBP^b^	123.5 (110–148)	147 (130-167.5)	113 (109–119)	< 0.001**
DBP^b^	79 (73–88)	86 (79–94)	76 (72–79)	< 0.001**

*ASCVD* Atherosclerotic cardiovascular disease, *HCs* Healthy controls, *NA* Not applicable, *SBP* Systolic blood pressure, *DBP* Diastolic blood pressure.

^a^Frequency with percentage for categorical variables.

^b^Mean with standard deviation (SD) or median with interquartile range (IQR) for continuous variables.

**p < 0.001.

### Clinical characteristics of ASCVD patients

**Fig. 2:**
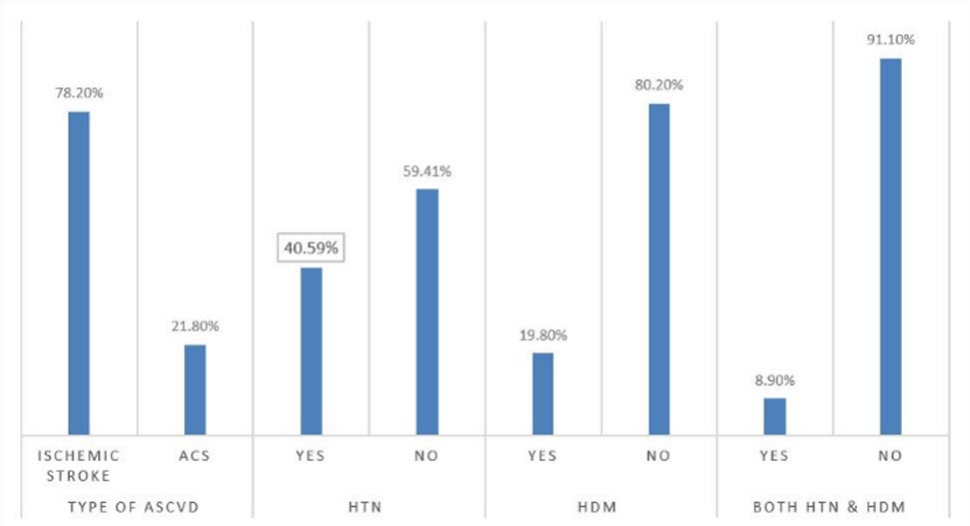
Clinical characteristics of ASCVD patients. Among 101 ASCVD patients, 78% had ischemic stroke and 22% had ACS. The clinical variables of ASCVD patients were shown on the X-axis with their corresponding percentage on the Y-axis. *HTN* History of hypertension, *HDM* History of diabetes mellitus, *ACS* Acute coronary syndrome.

**Fig. 3:**
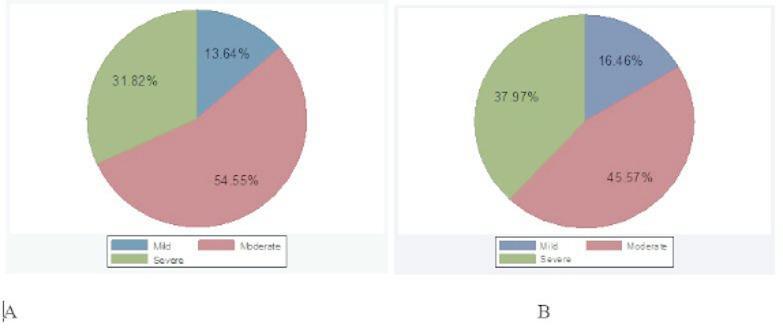
Severity manifestations of ASCVD patients. Among 22 ACS patients, 54.55% had moderate severity (represented in purple), while 45.57% of 79 ischemic stroke patients also showed moderate severity (also in purple). Part (**A**) represents severity among ACS patients, while part (**B**) shows severity among ischemic stroke patients.

Out of the 101 ASCVD patients, 22 were ACS patients, while the remaining 79 were ischemic stroke patients. Among ASCVD patients, 40.59% and 19.80% had a history of hypertension and DM, respectively. While 9% (8.9%) had both hypertension and DM history (Fig. 2). Moreover, the majority of ASCVD patients, 54.55% of those with ACS and 45.57% of those with stroke, were classified as having a moderate severity status (Fig. 3).

### Distribution of inflammatory cells, inflammatory cells ratio, hsCRP, and lipid profile among study participants

The mean level of WBC (8242 cells/µL versus 5784 cells/µL, *p* < 0.001) and neutrophil counts (5823 cells/µL versus 3453 cells/µL, *p* < 0.001), as well as the medians of monocytes (640 cells/µL versus 380 cells/µL, *p* < 0.001), were significantly higher in the ASCVD patients compared to HCs. However, the median of eosinophils (20 cells/µL versus 120 cells/µL, *p* < 0.001) was lower in ASCVD patients as compared to HCs (Table 2).

**Table 2:** Distribution of inflammatory cells, among the study participants from June to November 2024 at UoG-CSH Northwest Ethiopia.

Variable	ASCVD (*N* = 101))	HCs (*N* = 101)	*p*-value
WBC (cells/µL)^a^	8241.7 ± 1904.96	5784 ± 1763.43	< 0.001**
Neutrophil (cells/µL)^a^	5822.86 ± 1731.67	3452.673 ± 1535.042	< 0.001**
Lymphocyte (cells/µL)^a^	1618.81 ± 686.37	1678.02 ± 473.28	0.4763
Monocyte (cells/µL)^b^	640 (510–801)	380 (290–560)	< 0.001**
Eosinophil (cells/µL)^b^	20 (10–100)	120 (50–240)	< 0.001**
Basophil (cells/µL)^b^	10 (0–30)	20 (10–30)	0.0696
Platelet (cells/µL)^a^	243,604 ± 62008.56	238960.4 ± 43338.65	0.5381

*ASCVD* Atherosclerotic cardiovascular disease, *HC* healthy Controls, *WBC* White blood Cell.

^a^Mean with standard deviation (SD) using independent sample t-test.

^b^Median with interquartile range (IQR) using Mann-Whitney U test.

**p < 0.001.

**Fig. 4:**
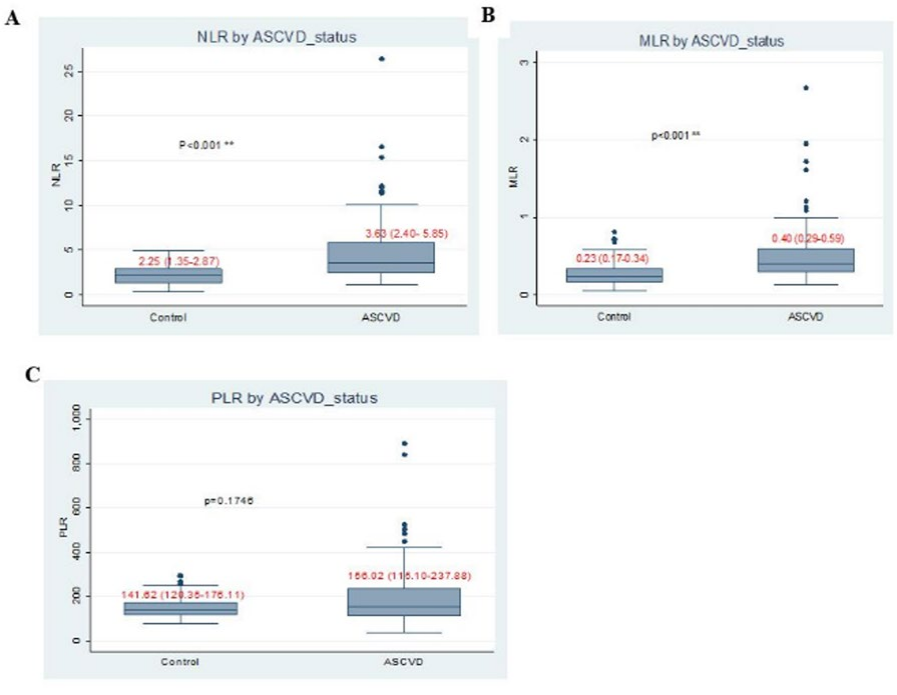
Distribution of the inflammatory cells ratio among the study participants at the UoG-CSH, Northwest Ethiopia. (**A**) The distribution of NLR. (**B**) The distribution of MLR, and (**C**) the distribution of PLR among ASCVD cases and healthy controls.

The median levels of NLR (3.63 versus 2.25, *p* < 0.001), MLR (0.40 versus 0.23, *p* < 0.001), and hsCRP (19.79 mg/L versus 0.93 mg/L, *p* < 0.001) were significantly higher in ASCVD patients compared to the HCs as shown in Fig. 4.

In addition, the level of TC (190 mg/dL vs. 170.6 mg/dL, *p* < 0.001) and LDL (122 mg/dL versus 101 mg/dL, *p* < 0.001) were significantly higher in ASCVD patients than HCs (Table 3).

**Table 3:** Distribution of hsCRP, and lipid profile among the study participants at the UoG-CSH, Northwest Ethiopia.

Variable	ASCVD (*N* = 101))	HCs (*N* = 101)	*p*-value
hsCRP (mg/L)^a^	19.79 (3.07–61.92)	0.93 (0 0.52- 2.19)	< 0.001**
TC (mg/dl)^a^	190 (173.8–220.4)	170.6 (151.6–194.6)	< 0.001**
TG (mg/dl)^a^	118 (91–148)	118 (91–143)	0.7644
LDL (mg/dl)^a^	122 (106.3–149)	101 (84–120)	< 0.001**
HDL (mg/dl)^b^	43.32 ± 13.07	45.75 ± 8.94	0.1249

*ASCVD* Atherosclerotic cardiovascular disease, *HCs* healthy Control, *hsCRP* High-Sensitivity C-Reactive Protein, *TC* Total cholesterol, *TG* Triglyceride, *LDL* Low density protein, *HDL* High density lipoprotein.

^a^Median with interquartile range (IQR) using Mann-Whitney U test.

^b^Mean with standard deviation (SD) using independent sample t-test.

**p < 0.001.

### Distribution of the inflammatory cells, inflammatory cells ratio, hsCRP, and lipid profile among ASCVD groups and healthy controls

To observe the impact of a history of hypertension (HTN) and diabetes mellitus (HDM) on ASCVD patients, a subgroup analysis was done by categorizing ASCVD patients into four groups: ASCVD with both hypertension and DM (HTN^+^HDM^+^), ASCVD with hypertension only (HTN^+^HDM^−^), ASCVD with DM only (HTN^−^HDM^+^), and ASCVD without HTN and DM (HTN^−^HDM^−^). The mean level of WBC and neutrophil count was significantly higher in all ASCVD groups compared to HCs, regardless of HTN and HDM. The median monocyte count was significantly higher in ASCVD HTN^+^HDM^+^ (*p* = 0.005), ASCVD with HTN^+^HDM^−^, and ASCVD with HTN^−^HDM^−^ (*p* < 0.001) compared to HCs. In contrast, the median eosinophil count was significantly lower in ASCVD with HTN^+^HDM^−^ (*p* = 0.006) and ASCVD with HTN^−^HDM^−^ (*p* = 0.009) compared to HCs (Table 4).

**Table 4:** Distribution of inflammatory cells among ASCVD groups and healthy controls at the UoG-CSH, Northwest Ethiopia.

Variable	ASCVD HTN^+^ & HDM^+^ (*N* = 9)	ASCVD HTN^+^ & HDM^−^ (*N* = 32)	ASCVD HTN^−^ & HDM^+^ (*N* = 11)	ASCVD HTN^−^ & HDM^−^ (*N* = 49)	HCs (*N* = 101)	*p*-value
WBC (cells/µL)^a^	7215 ± 2453.87	8521.60 ± 1727.14	7638.43 ± 2105.56	8252.77 ± 1927.20	5784.00 ± 1763.43	p^a^ = 0.006* p^b^ < 0.001**
Neutrophil (cells/µL)^a^	4670 ± 2125.39	6088.57 ± 1558.37	5032.71 ± 1479.30	5882.26 ± 1789.82	3452.67 ± 1535.04	p^a^ = 0.003* p^b/c/d^ < 0.001**
Lymphocyte (cells/µL)^b^	1825 (1250–1950)	1650 (1080–2090)	1610 (1400–2920)	1570 (950–2070)	1730 (1270–1970)	NS
Monocyte (cells/µL)^b^	850 (610–870)	720 (510–810)	600 (400–640)	630 (500–800)	380 (290–560)	p^a^=0 0.042* p^b/d^ < 0.001**
Eosinophil (cells/µL)^b^	50 (10–80)	20 (10–100)	20 (0–90)	40 (10–190)	120 (50–240)	p^b^ = 0.006* p^d^ = 0.009*
Basophil (cells/µL)^b^	0 (20–30)	0 (10–30)	0 (10–70)	0(10–30)	20 (10–30)	NS
Platelet (cells/µL)^b^	286,500 (257000–301000)	257,000 (196000–286000)	227,000 (174000–280000)	249,000 (209000–288000)	232,000 (203000–276000)	NS

^a^Mean ± SD using ANOVA.

^b^Median ± IQR using Kruskal–Wallis test.

p^a^: P-value for ASCVD having both HTN and DM vs. heathy control, p^b^: P-value for ASCVD with hypertension only vs. heathy control, p^c^ :P-value for ASCVD with diabetes mellitus only vs. heathy control, p^d^ :P-value for ASCVD with neither condition vs. heathy control.

*HTN* history of hypertension, *HDM* History of diabetes mellitus. NS: there is no statically significant differences, * = *p* < 0.05, ** = *p* < 0.001.

The median NLR was significantly higher in ASCVD with HTN^+^HDM^−^ and HTN^−^HDM^−^ (*p* < 0.001) and ASCVD with HTN^−^HDM^+^ (*p* = 0.042) as compared to HCs. Also, the median MLR was significantly higher in ASCVD with HTN^+^HDM^−^ and HTN^−^HDM^−^ (*p* < 0.001) as compared to HCs. Moreover, the median level of hsCRP in all groups of ASCVD, regardless of HTN and HDM, was significantly higher as compared to HCs. The median levels of TC and LDL were also significantly higher in ASCVD with HTN^+^HDM^−^ (*p* = 0.05 & 0.001, respectively) and ASCVD HTN^−^HDM^−^ (*p* = 0.006 & < 0.001, respectively) as compared to HCs. However, the median of HDL was significantly lower in ASCVD with HTN^+^HDM^+^ than in ASCVD with HTN^−^HDM^−^ (*p* = 0.033) and HCs (*p* = 0.021) (Table 5).

**Table 5:** Distribution of inflammatory cells ratio, and HsCRP among ASCVD groups and healthy control at the UoG-CSH, Northwest Ethiopia.

Variable	ASCVD HTN^+^ & HDM^+^ (*N* = 9)	ASCVD HTN^+^ & HDM^−^ (*N* = 32)	ASCVD HTN^−^ & HDM^+^ (*N* = 11)	ASCVD HTN^−^ & HDM^−^ (*N* = 49)	HC (*N* = 101)	*p*-value
NLR^a^	3.7 (2.04–5.40)	3.66 (2.79–4.88)	4.28 (2.13–4.67)	3.28 (2.38–6.56)	2.25 (1.35–2.87)	p^b/d^ < 0.001** p^c^ = 0.042*
MLR^a^	0.43 (0.31–0.49)	0.475 (0.31–0.64)	0.31 (0.25–0.46)	0.4 (0.29–0.58)	0.23 (0.17–0.34)	p^b/d^<0.001**
PLR^a^	152.94 (128.10–216.70)	163.7 (112.00–260.05)	140.99 (121.74–191.15)	151.49 (114.01–237.88)	141.62 (120.35–176.11)	NS
hsCRP (mg/L)^a^	30.71 (20.35–94.26)	4.92 (2.22–64.72)	27.31 (0.98–71.93)	42.22 (4.48–59.38)	0.93 (0.52–2.19)	p^a/b/d^ < 0.001** p^c^ = 0.005*
TC (mg/dl)^a^	186.7 (166.6–229.2)	192.8 (173–227.6)	185.4 (166.8–220.4)	189.6 (179–209.6)	170.6 (152–194.6)	p^b^ =0.05* p^d^ =0.006*
TG(mg/dl)^a^	153 (122–176)	118 (96–163)	131 (104–210)	108 (85–132)	118 (91–143)	NS
LDL(mg/dl)^a^	124.5 (106–162)	127 (114–155)	119 (102–145)	117 (105–147)	101.3 (84–120)	p^b^ = 0.001* p^d^ < 0.001**
HDL(mg/dl)^a^	28.5 (21–43)	43 (34–48)	42 (41–44)	47 (41–52)	44 (41–50)	p^e^ = 0.033* p^a^ = 0.021*

^a^Median ± IQR using Kruskal–Wallis test.

P^a^ :P-value for ASCVD having both HTN and DM vs. heathy control, P^b^ :P-value for ASCVD with hypertension only vs. heathy control. P^c^ :P-value for ASCVD with diabetes mellitus only vs. heathy control, P^d^ :P-value for ASCVD with neither condition vs. heathy control, p^e^ :P-value for ASCVD with both condition vs. ASCVD with neither condition.

*NS* there is no statically significant differences.

*p < 0.05, **p < 0.001.

## Discussion

In this study, 202 participants were included, comprising 101 newly diagnosed ASCVD patients and 101 healthy controls (HCs). Males presented the majority of ASCVD patients (52.5%), which is consistent with a study conducted at Belagavi India^35^, Dessie Comprehensive Specialized Hospital^16^, showing that men have a higher predisposition to ASCVD, potentially due to hormonal differences, higher prevalence of smoking, alcohol consumption, and occupational stress^36^. The mean (± SD) age of ASCVD patients was 57.42 ± 12.88 years, indicating that most ASCVD patients fell within the middle-aged to older adult range, which might be due to cumulative exposure to risk factors and age-related vascular changes^37^.

This study demonstrated that certain inflammatory markers, such as white blood cell (WBC) counts, neutrophils, monocytes, NLR, MLR, hsCRP, as well as TC, and LDL were significantly higher while eosinophil count was lower in ASCVD patients compared to HCs.

The observed higher neutrophil counts in ASCVD patients compared to healthy controls (*p* < 0.001) might indicate that neutrophils cause the development of ASCVD^38^. Neutrophils play a direct role in the development of atherosclerosis through the process of NETosis and secretion of inflammatory mediators such as MPO and oxygen-free radicals^39^. Ox-LDL causes the formation of NETosis through the activation of TLR-PKC-IRAK-MAPK and NADPH-oxidase^40^. NETosis can activate macrophages, leading to the amplified production of pro-inflammatory cytokines such as TNF-α and IL-6^41^. This inflammatory cascade might intensify the immune response, promoting the recruitment of additional immune cells (macrophages) to atherosclerotic plaques, causing plaques to rupture^42^, and accelerating disease progression^43^. The released MPO also causes further oxidation of LDL and HDL^44^ recruitment of the inflammatory cells and apoptosis (increase infarction)^45^. Those findings were in line with previous findings from China^46^, Ukraine^47^, Yemen^30^, and Jimma^48^.

The current study also demonstrated a significantly elevated monocyte counts in ASCVD patients compared to healthy controls (*p* < 0.001). This findings underscore the critical role of monocytes in the development of ASCVD^49^. Monocytes, upon infiltrating the vascular wall, differentiate into macrophages or dendritic cells, contribute to foam cell formation^22,50^ by recognizing oxLDL through SR-A1 and CD-36, and remain retained on the atherosclerotic plaques, driving the progression of atherosclerotic lesions^51^. Also, the recruitment of monocytes to the vascular wall may cause the vulnerability of plaques; this may be because of the production of inflammatory cytokines and MMPs from proliferated macrophages^52^. Previous study findings from China^46^, Ukraine^47^, and Yemen^30^ were also consistent with the current findings.

Interestingly, our study revealed significantly higher eosinophil counts in apparently healthy controls than in ASCVD patients (*p* < 0.001). The protective role of eosinophils could plausibly explain this, as they appear to be negatively correlated with the development of ASCVD^53,54^. For instance, eosinophils may improve cardiac function following myocardial infarction^55^, and low eosinophil count is associated with myocardial infarction mortality^56^. This is because eosinophils significantly blocked H_2_O_2_-induced cardiomyocyte death through their IL-4 secretion, inhibiting leukocyte adhesion and accumulation^55^. Furthermore, eosinophil cationic proteins (ECPs) are anticipated to regulate the polarization of macrophages and monocytes, as well as inhibit NF-κB activation in aortic inflammatory and vascular cells^57^. Lower eosinophil counts cause an increased lesion inflammatory cell content, matrix-degrading protease activity, cell proliferation, and apoptosis^58^.

Studies conducted in London^54^ and Jimma^48^ also consistent with this finding. However, this finding was not consistent with studies from China^59^ and the UK Biobank^60^, which reported that eosinophil count was a significant risk factor for ASCVD and its adverse outcome. This discrepancy might be due to differences in the study population and methodology.

This study also demonstrated the variations in NLR, MLR, and PLR between ASCVD patients and healthy controls, revealing significantly higher NLR and MLR levels in the ASCVD patients as compared to healthy controls (*p* < 0.001). The increased NLR and MLR in ASCVD patients may reflect heightened systemic inflammation, resulting in elevated neutrophil and monocyte counts^61^ and a reduction in lymphocyte counts due to physiological stress^62^. Additionally, IFN-γ-stimulated neutrophils may suppress lymphocyte proliferation by upregulating programmed death ligand 1 (PD-L1)^63^. Those findings were in accordance with studies from Yemen^30^, Turkey^64^, China^46,65,66^, India^67^, and Jimma^48^.

In current study, we observed a significant elevation in hsCRP, a sensitive marker of systemic inflammation in ASCVD patients compared to healthy controls (*p* < 0.001). This also underscores the strong inflammatory nature of ASCVD. Several studies suggested that CRP acts as a significant risk factor for ASCVD pathogenesis^68–70^. This is because CRP may influence multiple stages of atherosclerosis^28,71^. Specifically, it may directly impact processes such as complement activation, apoptosis, vascular cell activation, and enhanced expression of VCAM-1, reactive oxygen species (ROS) production, lipid accumulation, and thrombosis^29^. Additionally, CRP can induce leukocytes to release MMP^72^, which degrades elastin fibers in the arterial wall^73^, contributing to plaque instability^74^. Our findings are in agreement with the findings of research from India^12,13^.

Our study also revealed significantly elevated TC and LDL levels in ASCVD patients compared to healthy controls (*p* < 0.001). These findings reinforce the well-established role of elevated LDL cholesterol as a major risk factor for ASCVD^75^. This is because elevated LDL cholesterol contributes to endothelial barrier dysfunction^76^ and atherosclerosis development after crossing the endothelial walls with the help of scavenger receptor (SR-B1)^77^. Due to enzymatic (MPO) and non-enzymatic processes^78^, LDL undergoes oxidation to form oxLDL that will develop into foam cells that cause plaque formation^79^, while higher TC levels reflect the overall lipid burden associated with ASCVD. This finding is in accordance with previous results from studies in India^12,13^, Iran^11^, and Dessie Comprehensive Specialized Hospital^16^.

## Strengths and limitations of the study

As a strength of the study, we employed a comparative cross-sectional design, which enables a direct comparison of ASCVD patients and HCs regarding the level of inflammatory markers and lipid profile. In addition, we recruited newly diagnosed ASCVD cases within 72 h of ASCVD onset, which allows for early detection of inflammatory cells, hsCRP, and lipid profile levels with minimal influence from prolonged treatment and disease progression. However, we did not assess other inflammatory markers, such as inflammatory cytokines, which might be a good indicator of inflammatory response during ASCVD pathogenesis. The cross-sectional nature of the study did not allow for observing the causal relationship between inflammation, lipid profile, and the development of ASCVD. In addition, the study was conducted at a single center, which may limit the generalizability of the findings.

## Conclusion and recommendation

The current study demonstrated that levels of inflammatory cells (neutrophils and monocytes), NLR, MLR, and hsCRP, as well as lipid profiles TC and LDL, were significantly higher in ASCVD patients compared to HCs, highlighting the potential role of inflammation and dyslipidemia in the development of ASCVD. Based on our results, it is advisable to prioritize the prevention of chronic systemic inflammation and dyslipidemia to reduce the risk of ASCVD development. We also recommend conducting histopathological studies on atherosclerotic plaques or lesions obtained from autopsies to investigate inflammatory cell infiltration, lipid profile, and plaque stability, which could provide deeper mechanistic insights into the disease process.

## Supplementary Information

Below is the link to the electronic supplementary material.

Supplementary Material 1

Supplementary Material 2

## Data Availability

All the relevant data and supporting information files are within the manuscript.

## Abbreviations

ACS: Acute coronary syndrome
ASCVD: Atherosclerotic cardiovascular disease
CRP: C-reactive protein
CVD: Cardiovascular disease
EPHI: Ethiopian public health institution
H_2_O_2_: Hydrogen peroxide
HDL: High density lipoprotein
HDM: History of diabetes mellitus
hsCRP: High sensitive c-reactive protein
HTN: History of hypertension
LDL: Low density lipoprotein
MLR: Monocyte to lymphocyte ratio
MMP: Matrix metalloproteinase
MPO: Myeloperoxidase
NCD: Non-communicable disease
NLR: Neutrophil to lymphocyte ratio
Ox-LDL: Oxidized low-density lipoprotein
PLR: Platelet to lymphocyte ratio
ROS: Reactive oxygen specious
TC: Total cholesterol
TG: Triglyceride
UoG-CSH: University of Gondar Comprehensive Hospital
