# Supplementary material for: Comparison of inflammatory cells, C-reactive protein, and lipid profile in atherosclerotic cardiovascular disease patients and healthy controls in Northwest Ethiopia
Source: Sci Rep. 2025 Oct 27;15:37448. doi: 10.1038/s41598-025-21319-5 (PMC12559173; doi:10.1038/s41598-025-21319-5)
Supplement: Supplementary file 1 — Supplementary Material 1 [file 41598_2025_21319_MOESM1_ESM.pdf]

S1 Table: Variable assessment questionnaire

**Socio-demographic characteristics of study participants**		
**1**	Sex	1. Male 2. Female
**2**	Age (in year)	____________
**3**	Residence	1. Urban 2. Rural
**4**	Marital status	1. Single: 2. Married:<br>3. Divorced: 4. Widowed:
**5**	Educational status	1. Not have formal education at all<br>2. Primary<br>3. Secondary<br>4. Collage and above
**6**	Smoking status	1. Never 2. Yes
**7**	Alcohol consumption status	1. Never 2. Yes
**8**	Physical exercise habit	1. No/Sedentary<br>2. Yes
**9**	Family history of Atherosclerotic Cardiovascular Disease (ASCVD)	1. No<br>2. Yes
**Clinical characteristics of study participants**		
**1**	Systolic blood pressure	___________mmHg

**2**	Diastolic blood pressure	____________mmHg
**3**	History of Hypertension	1. Yes 2 .No
**4**	History of DM	1. Yes 2.No
**5**	Type of ASCVD	1. ACS<br>2. Ischemic stroke
**6**	Severity of ASCVD:	1. Mild<br>2. Moderate<br>3. Severe

Laboratory analysis of study participants

**Hematological Profile**		
**1**	Total white blood cell	_______________ Cells/µl
**2**	Neutrophil	_______________ Cells/µl
**3**	Lymphocyte	_______________ Cells/µl
**4**	Monocyte	_______________ Cells/µl
**5**	Eosinophil	_______________ Cells/µl
**6**	Basophil	______________ Cells/µl
**7**	Platelets	______________ Cells/µl
**Lipid profile and hsCRP**		
**1**	Total cholesterol	______ mg/dl
**2**	Triglyceride	_______ mg/dl

**3**	Low density lipoprotein (LDL)	________ mg/dl
**4**	High density lipoprotein (HDL)	________ mg/dl
**5**	High sensitive CRP (hsCRP)	________ mg/dl
