# Supplementary material for: Comparison of inflammatory cells, C-reactive protein, and lipid profile in atherosclerotic cardiovascular disease patients and healthy controls in Northwest Ethiopia
Source: Sci Rep. 2025 Oct 27;15:37448. doi: 10.1038/s41598-025-21319-5 (PMC12559173; doi:10.1038/s41598-025-21319-5)
Supplement: Supplementary file 2 — Supplementary Material 2 [file 41598_2025_21319_MOESM2_ESM.docx]

**S1 Checklist: STROBE checklist for observational study**

|  | Item No. | Recommendation | Page No | Relevant text from manuscript |
| --- | --- | --- | --- | --- |
| Title and abstract | 1 | (a) Indicate the study’s design with a commonly used term in the title or the abstract | 2 | An institution-based comparative cross-sectional study |
|  |  | (b) Provide in the abstract an informative and balanced summary of what was done and what was found | 2 |  |
| Introduction | | | |  |
| Background/rationale | 2 | Explain the scientific background and rationale for the investigation being reported | 3-4 |  |
| Objectives | 3 | State specific objectives, including any prespecified hypotheses | 4 |  |
| Methods | | | |  |
| Study design | 4 | Present key elements of study design early in the paper | 5 |  |
| Setting | 5 | Describe the setting, locations, and relevant dates, including periods of recruitment, exposure, follow-up, and data collection | 5 | ASCVD patients at the University of Gondar Comprehensive Specialized Hospital June to November 2024 |
| Participants | 6 | Cross-sectional study—Give the eligibility criteria, and the sources and methods of selection of participants | 5-6 |  |
| Variables | 7 | Clearly define all outcomes, exposures, predictors, potential confounders, and effect modifiers. Give diagnostic criteria, if applicable |  | NA |
| Data sources/ measurement | 8* | For each variable of interest, give sources of data and details of methods of assessment (measurement). Describe comparability of assessment methods if there is more than one group |  | NA |
| Bias | 9 | Describe any efforts to address potential sources of bias |  | NA |
| Study size | 10 | Explain how the study size was arrived at | 6 | The sample size was calculated by using G-power software with t-test family, A two-tailed, a power of 80%, α-error probability of 0.05, and 40% effect size. The total sample size was 202 (101 participants in each group). |

| Quantitative variables | 11 | Explain how quantitative variables were handled in the analyses. If applicable, describe which groupings were chosen and why | 7-9 |  |
| --- | --- | --- | --- | --- |
| Statistical methods | 12 | (a) Describe all statistical methods, including those used to control for confounding | 10 |  |
|  |  | (b) Describe any methods used to examine subgroups and interactions | 10 |  |
|  |  | (c) Explain how missing data were addressed |  | NA |
|  |  | (d) Cross-sectional study—If applicable, describe analytical methods taking account of sampling strategy |  | NA. |
|  |  | (e) Describe any sensitivity analyses |  | NA |
| Results | | | | |
| Participants | 13* | (a) Report numbers of individuals at each stage of study—eg numbers potentially eligible, examined for eligibility, confirmed eligible, included in the study, completing follow-up, and analysed | 11-12 | 202 study participants with 101 newly diagnosed ASCVD patients and 101 healthy controls (HCs) and from those 52.5% of both ASCVD patients and HCs were males. The mean with SD age of study participants was 57.42 ± 12.88 and 56.30 ± 10.93 years for ASCVD patients and HCs, respectively |
|  |  | (b) Give reasons for non-participation at each stage |  | NA |
|  |  | (c) Consider use of a flow diagram |  | NA |
| Descriptive data | 14* | (a) Give characteristics of study participants (eg demographic, clinical, social) and information on exposures and potential confounders | 11-12 |  |
|  |  | (b) Indicate number of participants with missing data for each variable of interest |  | NA |
| Outcome data | 15* | Cross-sectional study—Report numbers of outcome events or summary measures | 12-13 |  |
| Main results | 16 | (a) Give unadjusted estimates and, if applicable, confounder-adjusted estimates and their precision (eg, 95% confidence interval). Make clear which confounders were adjusted for and why they were included |  | NA |
|  |  | (b) Report category boundaries when continuous variables were categorized |  | NA |
|  |  | (c) If relevant, consider translating estimates of relative risk into absolute risk for a meaningful time period |  | NA |

Continued on next page

| Other analyses | 17 | Report other analyses done—eg analyses of subgroups and interactions, and sensitivity analyses |  | NA |
| --- | --- | --- | --- | --- |
| Discussion | | | | |
| Key results | 18 | Summarise key results with reference to study objectives | 14-17 |  |
| Limitations | 19 | Discuss limitations of the study, taking into account sources of potential bias or imprecision. Discuss both direction and magnitude of any potential bias | 17 | We did not assessed other inflammatory marker such as inflammatory cytokines, which might be a good indicator of inflammatory responses during ASCVD pathogenesis. Also cross-sectional nature of study did not allow to observe the casual relationship of inflammation, lipid profile, and the development of ASCVD. |
| Interpretation | 20 | Give a cautious overall interpretation of results considering objectives, limitations, multiplicity of analyses, results from similar studies, and other relevant evidence | 18 |  |
| Generalisability | 21 | Discuss the generalisability (external validity) of the study results | 18 |  |
| Other information | |  | | |
| Funding | 22 | Give the source of funding and the role of the funders for the present study and, if applicable, for the original study on which the present article is based | 18 |  |
